# Effects of gill and muscle biopsies on the short‐term behaviour, exercise performance and survival of juvenile lake trout (*Salvelinus namaycush*)

**DOI:** 10.1111/jfb.70343

**Published:** 2026-02-03

**Authors:** Laura Haniford, Connor H. Reid, Gillian Zorn, Graham D. Raby, Steven J. Cooke

**Affiliations:** ^1^ Department of Biology Carleton University Ottawa Ontario Canada; ^2^ Environmental and Life Sciences Graduate Program Trent University Peterborough Ontario Canada; ^3^ Department of Biology Trent University Peterborough Ontario Canada; ^4^ Institute of Environmental and Interdisciplinary Science Carleton University Ottawa Ontario Canada

**Keywords:** animal welfare, fish behaviour, lake trout, non‐lethal biopsy, physiological assessment, telemetry

## Abstract

Non‐lethal biopsy is a valuable tool for gaining insight into the physiological status of fish in the wild and for predicting their subsequent behaviour and survival. However, linking the insights from biopsy to post‐release behaviour relies on the assumption that the biopsy itself has no meaningful impact on post‐release behaviour or survival (e.g. in animals tagged with electronic devices). This assumption is likely to be questioned by fishery managers, animal ethics committees, and other researchers. To date, there has been very little work to assess the sublethal (or lethal) effects of biopsies on fish, and no such studies have evaluated fine‐scale behaviours. Here, muscle and gill tissue samples were taken (both individually and as a combined treatment) from hatchery‐reared juvenile lake trout (*Salvelinus namaycush*) in captivity. Twenty‐four hours after sampling, we used two behavioural assays (behaviour within a Z maze and flight initiation distance) and quantified exhaustive exercise performance to determine whether biopsies impacted behaviour or physiology when compared to non‐sampled controls. We found no evidence that biopsies had any influence on exploratory and shelter‐seeking behaviour in the maze, flight initiation distance, or time to exhaustion (as a proxy for swimming performance). Mortality during a 7‐day monitoring period was very low (2% across treatments) and limited to fish that received either a gill biopsy or the combined biopsy treatment. This study provides empirical support for the use of non‐lethal biopsy in juvenile salmonids as a means of collecting physiological data on individuals in behavioural studies and experiments.

## 1 INTRODUCTION

Physiological biopsies (e.g. gill, muscle, or blood sampling) are increasingly used in fisheries science to assess stress, metabolic status, pathogen load, and contaminant exposure (Cooke et al., 2005; Jeffries et al., 2021). Biopsies provide a mechanism to reduce mortality (relative to lethal sampling), can allow for repeated sampling of individual fish over time, and can be used in laboratory or field experiments to link physiological state to endpoints like growth, behaviour, or survival. Biopsies are often paired with electronic tagging and tracking studies to link a fish's internal state to its movement, offering a valuable approach for identifying the processes which drive behaviour, fate, and survival (Thorstensen et al., 2022). As fisheries agencies increasingly rely on electronic tagging and tracking tools to inform management (Brooks et al., 2019; Lowerre‐Barbieri et al., 2019), pairing biopsies with such tagging represents an important next step in using these technologies to explain the variation in fish movement and performance.

Despite their potential benefits, the use of biopsies makes a key assumption: that taking the biopsy itself does not impact the fish and, by extension, confound the study. Most validation studies suggest that biopsies have little effect on adult fish survival or reproduction (Jeffries et al., 2014; McCormick, 1993). For example, blood and gill biopsies were not found to change survival or migration of sockeye salmon (Cooke et al., 2005), and individual gill, muscle, or blood biopsies did not affect nesting success in smallmouth bass (Haniford et al., 2023). However, not all research has supported this pattern. Gill biopsies were identified as a significant predictor of mortality in the first segment of migration for some juvenile sockeye salmon (Bass et al., 2020), suggesting that biopsy impacts may be more likely to manifest in small‐bodied species or early life stages. Such conflicting findings, even within the same species, highlight the need for further validation of biopsy impacts.

Currently, most field‐based validation studies have focused on survival and reproduction, providing limited resolution on short‐term behavioural responses following biopsy during the period when stress responses are most likely to occur. Although biopsies are considered minimally invasive, they can still cause localized tissue damage, inflammation, and temporary physiological alterations (Cooke et al., 2005; Jeffries et al., 2021; Thorstensen et al., 2022). Gill biopsies may impair ion regulation and gas exchange due to epithelial damage, and significant blood loss can occur when biopsies are taken from a highly vascularized area (Goss et al., 1998). Similarly, muscle biopsies can cause localized tissue damage, triggering inflammation, bruising and recruitment of immune cells (Bøe et al., 2020). Both biopsy types can disrupt the protective mucus layer and increase susceptibility to secondary infections (Jeffries et al., 2021; Thorstensen et al., 2022). While responses are usually mild and transient in healthy fish, individual variation in immune function or pre‐existing health conditions could influence resilience to biopsy and contribute to any behavioural differences observed post‐treatment (Cooke et al., 2005). Because many of the potential effects of biopsy (e.g. stress, inflammation, tissue damage) are likely to manifest first through changes in behaviour rather than overt mortality, short‐term behavioural assessments may provide an early and sensitive measure of biopsy's impact. Indeed, behaviour provides a sensitive and non‐invasive indicator of fish health and welfare, with deviations from normal behaviour (such as exploratory behaviour, ventilation, or foraging) often linked to poor health or condition (Ashley, 2007; Huntingford et al., 2006; Martins et al., 2012; Yi et al., 2021). Behavioural indicators are increasingly used in aquaculture and fish welfare research (Martins et al., 2012; Poli, 2009; Schreck et al., 1997), but their application to biopsy validation remains limited.

Here, we tested whether gill and muscle biopsies influence the behaviour, exercise performance, or survival in hatchery‐reared juvenile lake trout (*Salvelinus namaycush*). By combining exploratory behaviour, reactivity, and swimming assays with mortality monitoring, we directly address a knowledge gap related to understanding how non‐lethal biopsies influence fish after sampling. We tested the hypothesis that biopsy procedures would not alter the behaviour, survival, or performance of fish relative to controls, thus supporting their continued use as a minimally invasive sampling method in fish tracking and other experimental studies.

## 2 METHODS

All research conducted in this study was carried out following the Carleton University Animal Care Protocol (REF 110723), consistent with the fish care guidelines from the Canadian Council for Animal Care.

### 2.1 Fish collection and treatments

We used 128 juvenile lake trout (total length 171 ± 19 mm [mean ± standard deviation]) from the White Lake Fish Culture Facility in Sharbot Lake, ON, Canada (44.7699 N, 76.7472 W; an Ontario Ministry of Natural Resources hatchery used to produce fish to stock into lakes and rivers around the province). Fish were transferred by bucket from rearing tanks to flow‐through tanks that were gravity‐fed by a nearby lake, with a constant water temperature of 11°C. Six hours after transfer, fish were randomly assigned to one of five treatments: a gill biopsy, a muscle biopsy, a combined biopsy treatment (gill and muscle), a tagged control, and an untagged control, with *n* = 25 fish per treatment. Any fish that died during the 24‐h period between biopsy treatment and behavioural testing were replaced with another fish, which was given the same biopsy type to maintain balanced sample sizes for statistical analyses. All fish were briefly air‐exposed when measured for total length and weight to ensure sizes were consistent between treatments. Fish from the biopsy treatments and the tagged control group were tagged using 8‐mm passive integrated transponder (PIT) tags for individual identification, which were inserted immediately after the fish were measured for length and weight. Tags were inserted in a small incision of approximately 2 mm, made using a scalpel, halfway between the anus and pelvic fins. All PIT tags were cleaned using Betadine povidone iodine before insertion, and no Vetbond or sutures were used to close the incision due to its small size. Fish from the untagged control group were identified based on total length and weight measurements. No anaesthesia was used during fish measurements or biopsy.

**FIGURE 1:**
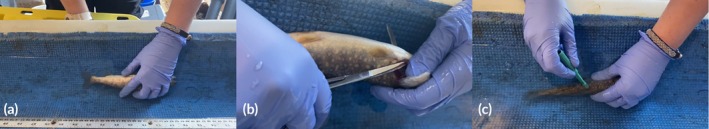
Experimental setup and biopsy procedures for juvenile lake trout (*Salvelinus namaycush*): (a) the setup for handling fish during biopsies, (b) the process of taking gill biopsies with fine nail scissors and (c) muscle biopsies taken using a muscle punch, 4 mm diameter.

All fish were held in a soft, padded sampling trough for 2 min to ensure handling time was standardized between treatments, with their gills kept fully submerged (Figure 1). For the gill biopsy, the head of the fish was briefly lifted above the water to take 3–4 mm of gill tissue from the distal end of gill filaments on the second gill arch using fine nail scissors. Muscle biopsies were taken from the dorsal musculature directly below the dorsal fin (Figure 1) using a 4‐mm biopsy punch (Integra Miltex Disposable Biopsy Punch).

### 2.2 Behavioural assays

Twenty‐four hours after biopsy, we assessed behaviour using two assays: behaviour within a Z maze and flight initiation distance (FID). Previous research has used Z mazes as a method for studying exploratory and shelter‐seeking behaviour in a novel environment (Chapman et al., 2010; Hlina et al., 2021; Ramsaran et al., 2021). FID provides an assessment of how a fish reacts to a new object, an approach by a predator, or the discovery of food in the wild (Hlina et al., 2021; Wilson & Godin, 2009). Both the Z‐maze response variables and FID involve behaviours that may be relevant to the fitness of hatchery fish released into the wild.

**FIGURE 2:**
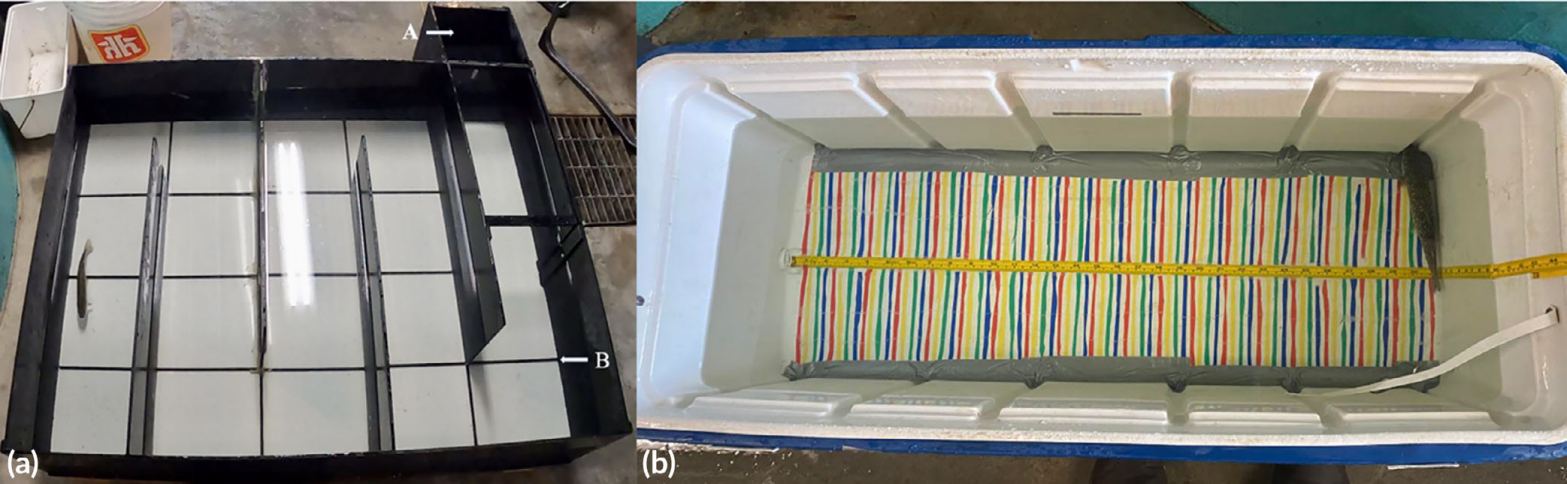
(a) Z maze, constructed out of black plexiglass and filled to a depth of 20 cm with fresh water. A refuge (A) is located at one end of the maze, which was gated and covered during the 5 minutes fish were given to acclimatize to the maze. A grid of 20 equal squares was created using lines of black plastic (B) laid along the bottom. (b) The flight initiation distance (FID) arena constructed using a cooler (80 × 30 cm) filled to a depth of 30 cm with fresh water. A measuring tape was fixed to the bottom of the cooler, with different colours of electrical tape marking each centimetre.

The Z maze we used was a rectangular arena constructed from black plexiglass, 100 × 80 cm, filled with fresh water from the flow‐through tanks to a depth of 20 cm, and had a gated and covered refuge (20 × 40 cm) at one end of the arena (Figure 2). Three opaque black partitions were used to divide the main area of the arena into a Z pattern. Black pieces of plastic were fixed to the bottom of the arena to create a grid of 20 equal squares, with four squares in each row of the maze. At the beginning of each trial, fish were introduced to the refuge and allowed 5 min to acclimatize to the space before the gate was opened, allowing the fish to explore for 15 min. A GoPro Hero 7 was mounted above the maze to record each behavioural trial, ensuring no human disturbance. Emergence from the refuge, the amount of time each fish spent in the refuge after the gate was opened, and activity level (total number of lines crossed, reflecting how actively fish swam around the arena) were all quantified from each 15‐min trial. The maze was drained, rinsed, and refilled with fresh water between trials.

Immediately after the Z‐maze trial, fish were transferred to the FID arena (an insulated tank, 80 × 30 cm, filled to a depth of 30 cm with a measuring tape lining the bottom and each centimetre marked in different colours of electrical tape; Figure 2). Fish were given 2 min to acclimatize to the FID arena before a novel object (a Carleton University Transit Pass resembling a credit card, 8.5 × 5.5 cm, light blue with holographic details on one side) was lowered into the water column at the far side of the arena from where the fish had settled with the holographic side facing towards the fish. The card was moved towards the fish's head at a rate of 1 cm s^−1^ (timed using a stopwatch). The approach stopped as soon as the fish moved to avoid the novel object, and the distance between the fish and the novel object was estimated (nearest cm) from the video recording.

### 2.3 Exhaustive exercise challenge

Following FID measurements, fish were transferred to a circular arena (diameter 100 cm, depth 20 cm) with a bucket (diameter 30 cm) placed in the centre to create a doughnut‐shaped track for forced exercise. Forced exhaustive exercise has been widely used as a simple means to assess the anaerobic swimming performance of fish (Clark et al., 2017; Kieffer, 2000; Milligan, 1996). Although this approach does not generate an estimate of maximum swimming speed like critical swimming speed (*U*_crit_), it does provide a useful approach for comparing groups of animals (Portz et al., 2006). Electrical tape was used to divide the arena into four equal quadrants to better determine the number of laps fish travelled around the chase tank. Fish were chased around the arena using a small hand net until they reached the point of exhaustion, classified as the point at which they failed to burst swim away from three consecutive tail‐grabs. The amount of time it took to reach the point of exhaustion for each fish and the number of laps fish swam were recorded for each individual using a hand‐held tally counter per Samson et al. (2014).

After the completion of assays, fish were returned to their holding tank, where their survival was monitored for 7 days. Next, the fish were euthanized using cerebral percussion, and the carcasses were examined for healing at the biopsy sites and for signs of disease. Any abnormal healing surrounding the biopsy site was noted, such as bruising and/or abscess formation. PIT tags were scanned to link injury with individual fish.

### 2.4 Statistical analysis

Data generated from this experiment were analysed using RStudio v. 4.1.2 (RStudio Team, 2021) with R v. 4.4.0 (R Core Team, 2021). Z‐maze behavioural metrics (successful emergence from the refuge, activity in the maze, and time spent in the refuge) were analysed as dependent variables, with the biopsy treatment treated as the independent variable. All data were visually assessed for assumptions using histograms and QQ plots. For statistics, the type I error rate (α) was set to 0.05.

Fish sizes were compared between treatment groups using an analysis of variance (ANOVA) to determine whether mean total length differed. A generalized linear model with binomial error distribution was used to compare emergence success among treatment groups. A zero‐inflated binomial model with a log‐link function was used to model the relationship between biopsy treatment and the number of grid lines crossed by fish in the Z maze. This model was selected due to both the presence of excessive zeros in the data (many fish did not leave the refuge, thus crossing zero grid lines) and overdispersion in the data. Time in the refuge, FID, and time to exhaustion were all non‐normally distributed, and no transformations could be applied to sufficiently correct for normality. Therefore, those data were analysed using a Kruskal–Wallis test, and the assumption of homogeneity of variance was assessed using a Fligner–Killeen test. Because all fish used in the study were juveniles from the same cohort and were of similar total length, FID was not scaled to body size. The number of laps around the chase tank was square root‐transformed to meet assumptions of normality and homogeneity of variance, and was analysed using a one‐way ANOVA.

## 3 RESULTS

**FIGURE 3:**
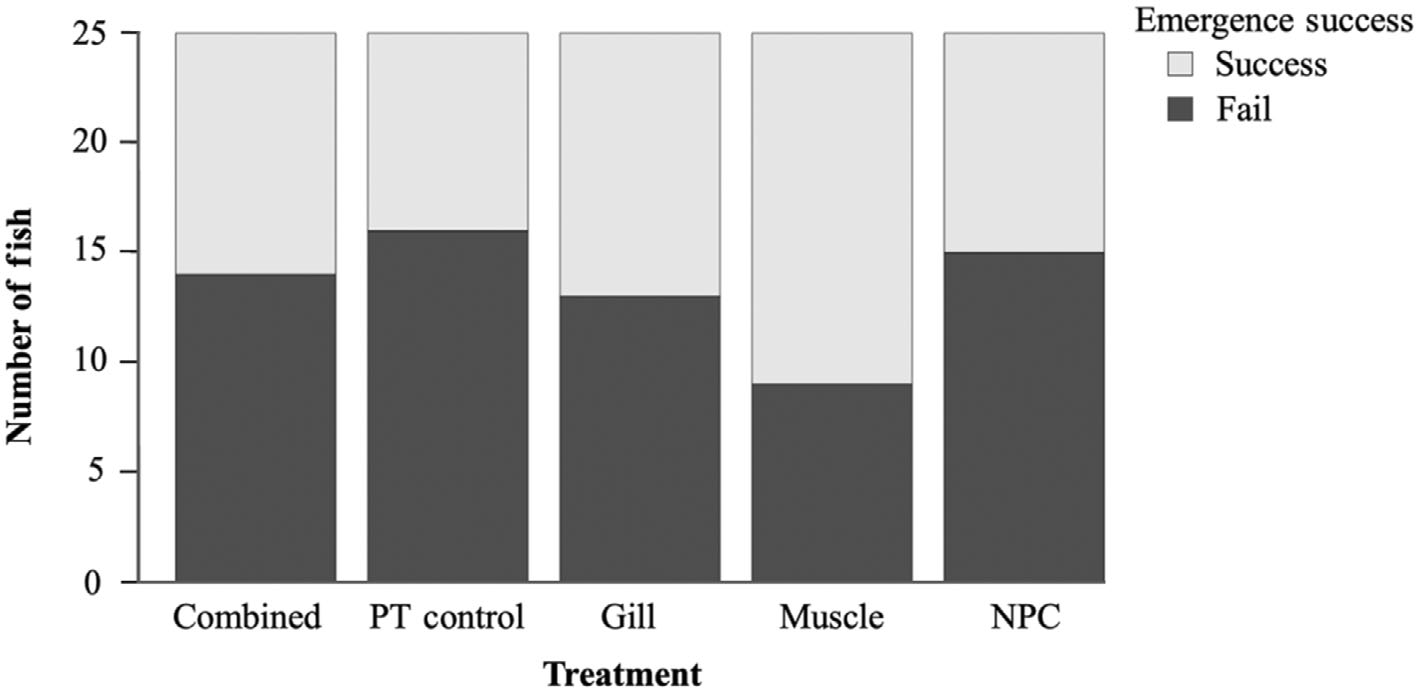
Successful emergence from the refuge by juvenile lake trout *(Salvelinus namaycush*) for each biopsy treatment type (*n* = 25 per treatment, combined = gill and muscle biopsy, PT control = pit‐tagged control, gill = gill biopsy, muscle = muscle biopsy, NPC = non‐pit‐tagged control) during the 15‐min exploratory window in the Z maze.

**FIGURE 4:**
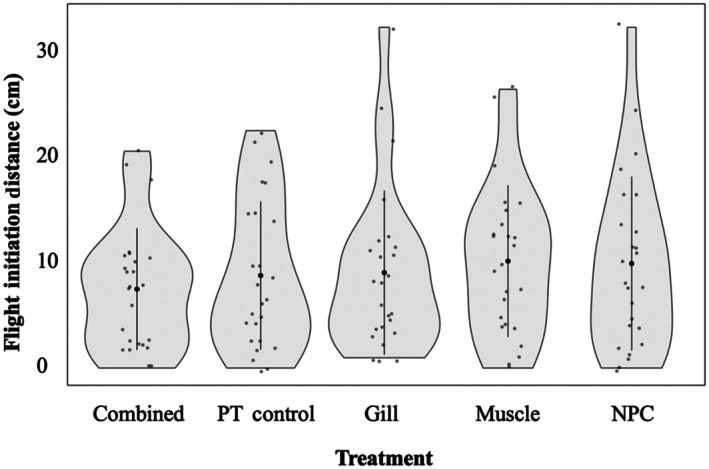
Flight initiation distance (in cm) measured in juvenile lake trout (*Salvelinus namaycush*) in response to a novel object compared across five different non‐lethal biopsy treatment types (*n* = 25 fish per treatment, combined = gill and muscle biopsy, PT control = pit‐tagged control, gill = gill biopsy, muscle = muscle biopsy, NPC = non‐pit‐tagged control). Black circles in the centre of each violin indicate the mean flight initiation distance (FID) value for each biopsy treatment group, with error bars showing the standard deviation, highlighting variability in behavioural responses.

**FIGURE 5:**
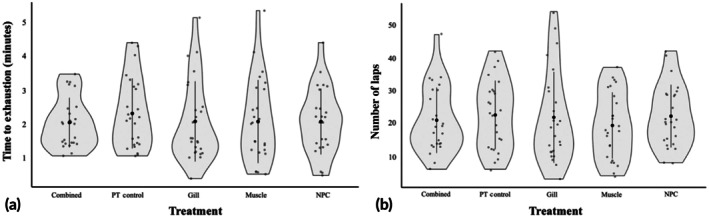
Comparison of swimming performance metrics for fish across the five different biopsy treatment groups, with *n* = 25 fish per treatment (combined = gill and muscle biopsy, PT control = pit‐tagged control, gill = gill biopsy, muscle = muscle biopsy, NPC = non‐pit‐tagged control). (a) Violin plot showing the time it took fish to reach the point of exhaustion (in minutes) during the exhaustive exercise assessment. (b) Violin plot showing the number of laps completed during the exhaustive exercise assessment, representing the activity levels across treatments. Black circles at the centre of each violin represent the mean value of that treatment group, with error bars showing standard deviation.

Average total length was consistent among biopsy types (*F* = 0.81, *df* = 4, *p* = 0.52). Emergence from the maze refuge was very low across treatments (Figure 3). Only 46% of fish emerged during the 15‐min trials. We found no evidence that biopsy treatment, regardless of type, affected the likelihood of emergence from the refuge (LR|^2^ = 4.72, *df* = 4, *p* = 0.32). The zero‐inflated Poisson model used to analyse the number of grid line crosses identified a significant intercept, indicating that there was a baseline level of inactivity throughout all fish, independent of biopsy type. Similarly, we found no evidence that biopsy treatment affected activity levels (Table 1) or time spent in the refuge (*H* = 5.57, *df* = 4, *p* = 0.11). Likewise, treatment did not affect FID (*H* = 2.00, *df* = 4, *p* = 0.74; Figure 4), time to exhaustion (*H* = 0.395, *df* = 4, *p* = 0.813), or the number of laps in the chase tank until exhaustion was achieved (*F* = 0.414, *df* = 4, *p* = 0.798; Figure 5).

**TABLE 1:** Summary of zero‐inflated model results for the number of grid lines crossed in the Z maze by each biopsy treatment group

(i)				
	Estimate	Standard error	*Z* value	*p* value
Intercept	4.5290	0.2944	15.383	2.00e−16***
Combined	0.2071	0.3867	0.536	0.5922
Pit‐Tagged control	−0.4223	0.4055	−1.041	0.2977
Gill	0.3051	0.3866	0.789	0.4299
Muscle	0.3805	0.3797	1.002	0.3162
Log(theta)	0.3829	0.1935	1.979	0.0478*

(ii)				
	Estimate	Standard error	*Z* value	*p* value
(Intercept)	0.7505	0.4292	1.749	0.0804
Combined	−0.5123	0.589	−0.87	0.3845
Pit‐Tagged Control	−0.1815	0.5989	−0.303	0.7618
Gill	−0.5119	0.589	−0.869	0.3848
Muscle	−0.6729	0.5872	−1.146	0.2518

*Note*: The table includes: (i) coefficients from the count model, representing the relationship between treatment and observed grid line counts, with the intercept representing the untagged control group, used as the reference group in the model coding, and log(theta) representing the dispersion parameter, and (ii) coefficients from the zero‐inflated model, describing predictors of excessive zeros. Asterisks indicate level of statistical significance, where *** denotes highly significant results, * denotes significant results, and no asterisk indicates a non‐significant result.

**FIGURE 6:**
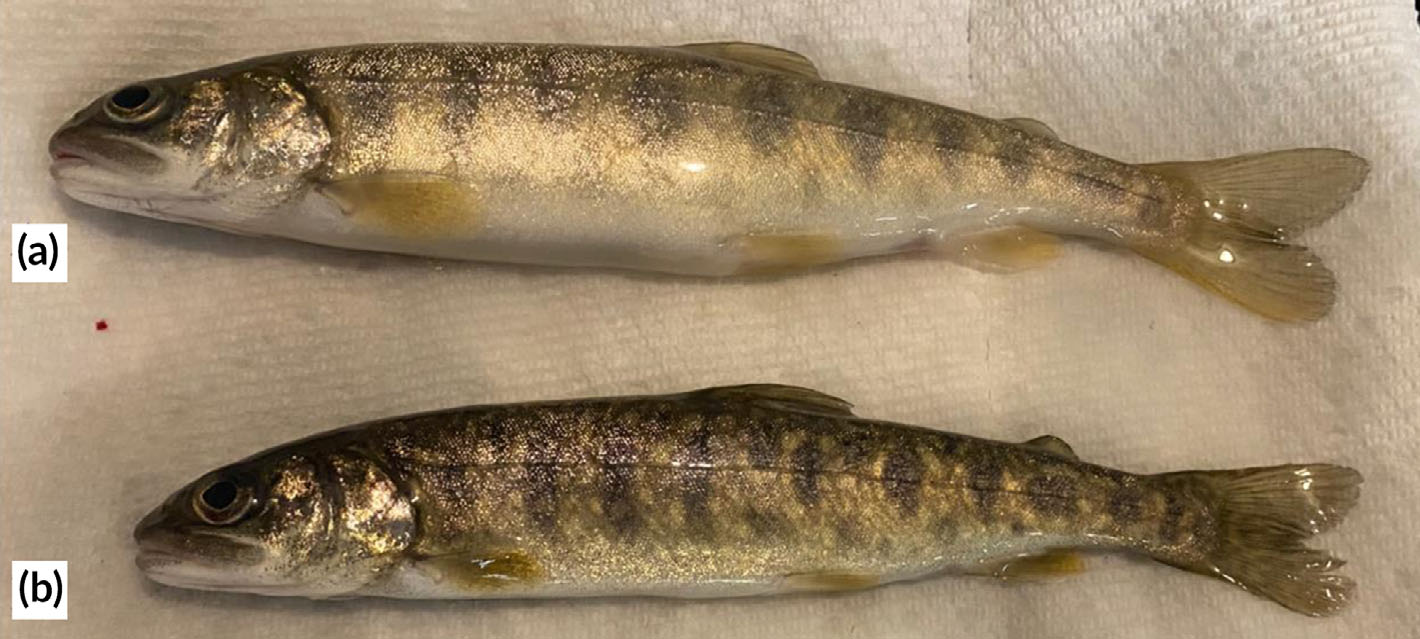
Juvenile lake trout (*Salvelinus namaycush*) pictured 4 h after non‐lethal gill biopsy; fish (a) was noticeably lighter in colour than fish (b), both of which underwent the same biopsy procedure (gill biopsy). Fish (a) was found dead the following day.

Mortality was very low throughout the study: only three mortalities occurred over 7 days (2% overall). All three mortalities occurred within 24 h after biopsy, with two fish coming from the gill biopsy treatment group and one fish coming from the combined biopsy treatment. External examination of the carcasses showed no apparent signs of bruising or disease, but the carcasses were lighter in colour than other fish sampled on the same day several hours after biopsies were taken (Figure 6). All fish that later died were seen respiring at the water's surface immediately after sampling; no other fish exhibited this behaviour.

**FIGURE 7:**
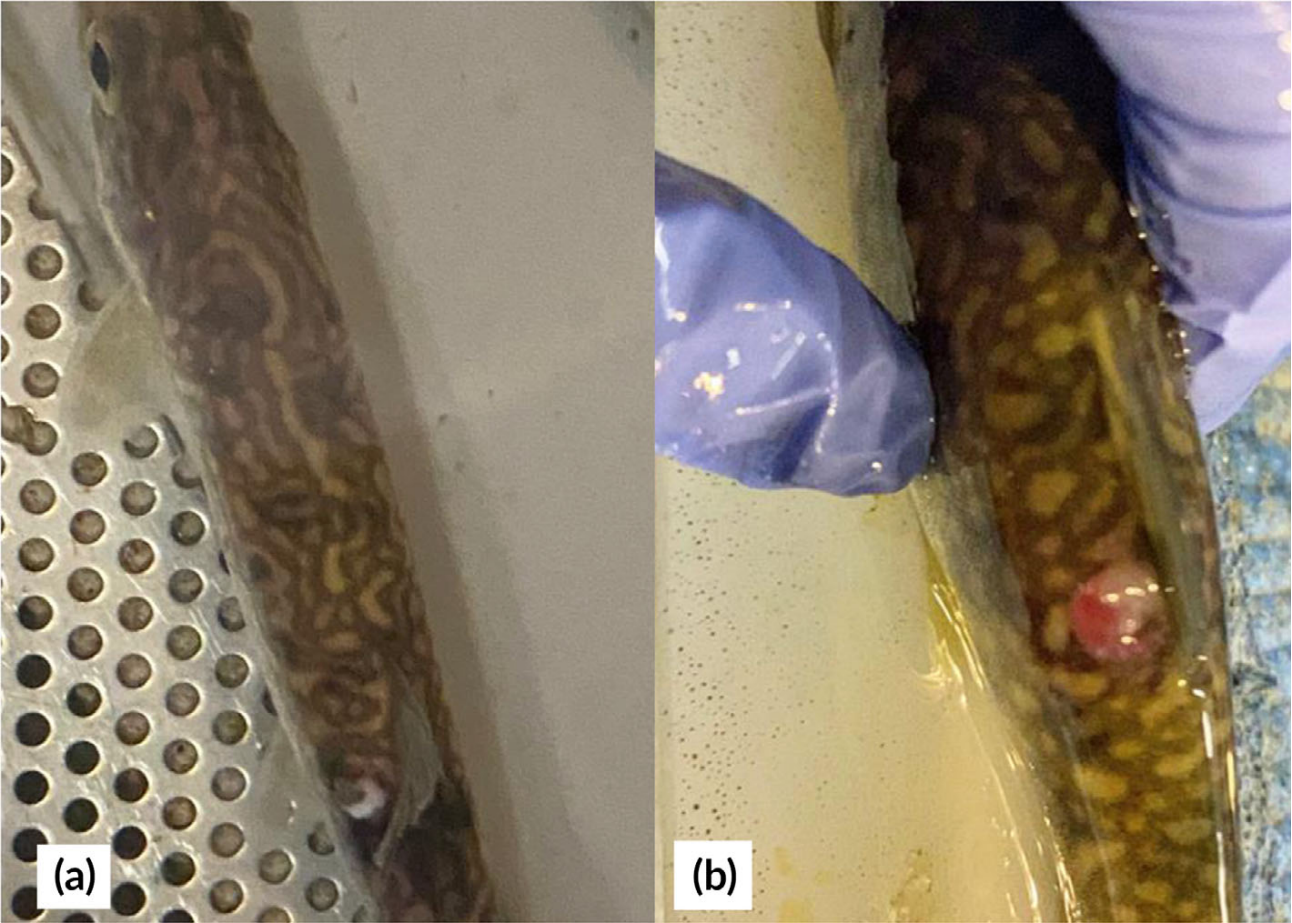
Juvenile lake trout (*Salvelinus namaycush*) photographed after dorsal muscle biopsies, showing post‐procedural outcomes noted in some fish. Significant bruising noted at the biopsy site (a) and an abscess noted at the biopsy site (b).

Evaluation of the biopsy sites after the 7‐day monitoring period revealed bruising at 23% of the muscle biopsy sites in fish from both the muscle and combined biopsy treatments, and abscesses at the muscle biopsy site in 8% of all fish that received muscle biopsies (Figure 7). Gill discolouration (light pink in colour, with pink or white tips of the gill filaments) was visible in 16% of all fish that received a gill biopsy, but not in fish from the control or muscle biopsy groups.

## 4 DISCUSSION

Based on our behavioural assays and an exhaustive exercise test conducted on 125 animals 24 h after handling, we found no evidence of biologically meaningful impacts of gill and muscle biopsy on juvenile lake trout, ultimately supporting our initial hypothesis. Mortality was negligible across treatments, and no treatment‐related effects were detected in our flight initiation distance, time to exhaustion, or number of laps swum during the exhaustive exercise challenge. Our ability to assess a range of behaviours within the Z maze was limited by a low rate of emergence from the refuge. However, the rate of emergence was consistent across treatments. In pilot experiments, we found that giving fish longer (up to 30 min) in the maze did not increase the rate of emergence from the refuge. Because all fish completed the behavioural trials in a fixed sequence (Z maze, followed by FID, then exercise challenge), we cannot fully separate the potential influence of trial order on behavioural outcomes. Nonetheless, the consistency of the results across treatments suggests that biopsy effects, if present, were minimal. These findings indicate that within the first 24 h post‐sampling, biopsy did not impair survival, physical performance, or exploratory behaviour.

One possible explanation for the overall low emergence rate observed in the Z maze is individual variation in fish personality. Previous work on behaviour in fishes (e.g., Réale et al., 2007; Wilson & Godin, 2009) documented a spectrum of personality, including a bold‐shy continuum; thinking about our findings in this context could help explain the low rate of emergence. Fish at the bold end of the continuum engage in a range of risk‐tolerant behaviours such as active exploration, whereas fish with a shy phenotype are more likely to seek or remain in shelter (Wilson et al., 1993). In the wild, boldness has been associated with the pace of life history, with bolder individuals typically predicted to grow faster but suffer higher rates of predation. In a hatchery environment where many fish come from the same broodstock and predation pressure is non‐existent, boldness may still be selected for during feeding, as more aggressive individuals are likely to eat more (Martins et al., 2012; Sundström et al., 2004). If we assume that behaviour is plastic, we would predict that more fish would fall on the shy end of the continuum in the hours or days following an injurious stressor (e.g. handling and biopsy). Emergence from a refuge is a classic assessment for boldness; we saw no evidence that fish were shifted towards the shy end of the behavioural spectrum as a result of our treatments. Consistently low emergence across treatments could have been attributed to handling stress, as opposed to biopsy, considering that all fish were handled at minimum to obtain length and weight measurements. Yet, air exposure was brief and water temperatures were cool such that handling stress would have been relatively low.

Similar to emergence from the refuge, exploratory swimming activity (in the context of the number of grid lines crossed in the maze) and time spent in the refuge were not found to relate to non‐lethal biopsy methods, although our sample sizes for these metrics were limited by low rates of emergence, which may have restricted our statistical power and ability to detect subtle behavioural differences. Although individual personality may further play a role in behaviours exhibited by fish and measured in this study, other research has found swimming activity to be a proxy for the stress status of fish (Svendsen et al., 2021). Our research found swimming activity throughout the Z‐maze trials to be consistent among biopsy treatments. As a result, we conclude that inter‐individual differences in behaviour in our trials likely resulted from intrinsic (personality) and genotypic/phenotypic differences rather than impacts of the biopsy.

Reactivity observed during the FID assays did not reveal differences between non‐lethal biopsy types, indicating that non‐lethal biopsy does not yield a significant enough amount of stress to impair reactivity or avoidance responses. Reactivity is an essential component of avoiding predation (Dill, 1974; Fuiman et al., 2006) and therefore plays a critical role in survival. Time to exhaustion and number of laps swum are similarly relevant to predator avoidance and unimpacted by our treatments. Collectively, the FID and exercise performance data suggest that by 24 h after release, biopsied fish should not be impaired in their ability to react to and escape from predators.

Minor tissue injuries caused by gill or muscle biopsy can initiate local inflammation and immune responses, which influence ion regulation, mucus production and overall stress physiology (Tort, 2011). Such physiological disturbances could, in theory, reduce swimming performance or alter behaviour through shifts in energy allocation or osmoregulatory balance. The absence of detectable differences in our behavioural and performance assays suggests that any inflammatory or stress responses elicited by biopsy were mild or rapidly resolved under our experimental conditions, consistent with other studies showing rapid recovery in salmonids (Cooke et al., 2005; Jeffries et al., 2014). Our findings align with previous work that has reported minimal effects of gill or muscle biopsies on fish survival, behaviour, or physiology (Henderson et al., 2016; McCormick, 1993: Cooke et al., 2005; Jeffries et al., 2014). Similar to our results, those studies observed rapid recovery of fish with negligible impacts on survival, performance or reproductive behaviours following non‐lethal sampling. Together, this growing body of evidence suggests that, when performed carefully, tissue biopsies pose little risk of compromising fish condition or performance.

Although mortality rates were low overall, previous research (e.g. Van Der Salm et al., 2004; Vitt et al., 2022) has shown that colour changes (as observed in fish in this experiment prior to mortality) can be reflective of the physiological state of the fish (Yi et al., 2021). While some fishes (e.g., gobies, cichlids) exhibit adaptive colour changes in response to their environment (Stevens et al., 2014), lake trout have limited capacity for plasticity in colouration. Instead, their colouration is more strongly influenced by genetics, diet, life history, and/or stress (Scott & Crossman, 1973; Zimmerman et al., 2006). For example, pigments such as carotenoids have been found to be directly affected by oxidative stress, which may be caused by challenges to the immune system (Sefc et al., 2014). Because stress‐related changes in pigment expression and concentration have been widely documented, colouration is increasingly recognized as an indicator of fish condition and welfare (Vitt et al., 2022). In our study, all three fish that died were lighter in colour than their counterparts as little as 4 h after biopsy. Similar colour changes were noted in the four fish from the muscle and combined biopsy treatments that had visible abscesses at the biopsy site 7 days later, when fish were examined at the conclusion of the holding period. Although not statistically meaningful, all three mortalities that did occur were exposed to gill biopsy. Further experiments with larger sample sizes and a wider range of species could be used to confirm whether gill biopsy could have subtle but important effects on post‐tagging survival. Collectively, these observations suggest a deterioration in health status likely associated with primary or secondary pathogen infection in a small subset (2%) of the biopsied fish.

Future studies could build upon this work by incorporating baseline assessments of animal personality prior to biopsy, which could help untangle treatment effects from variation in phenotypes. Investigating the time course of behavioural and physiological responses beyond the 24 h window used here could offer valuable insights, particularly regarding the recovery trajectories. Given the association between pigment changes and post‐biopsy mortality observed in a small subset of individuals, future work could explore whether these colour shifts are predictive of physiological stress or immune function. Finally, as all fish in this study were hatchery‐reared juveniles, future research should assess whether similar results may be found in wild fish, which may differ in stress responsiveness, pathogen load, or behavioural plasticity.

Ultimately, the results of this study suggest that non‐lethal biopsy methods do not affect the behaviour or performance of juvenile salmonids and are associated with negligible mortality. These findings support the use of biopsy as a tool for assessing physiological status without compromising behaviour, thus offering insights for future research on fish health and conservation. From a fisheries monitoring perspective, our results suggest that incorporating non‐lethal biopsy sampling into tagging and tracking programs is unlikely to bias subsequent behavioural data. By demonstrating that biopsied fish exhibit normal exploratory and performance behaviours within a 24‐h period post‐sampling, our findings indicate that these methods can yield unbiased insights into post‐tagging movement and habitat use.

## AUTHOR CONTRIBUTIONS

L.H.: Data collection, data analysis, preparation of manuscript. C.H.R. Data analysis, preparation of manuscript. G.Z.: Data collection, preparation of manuscript. G.D.R.: Project planning, preparation of manuscript. S.J.C.: Project planning, preparation of manuscript.

## FUNDING INFORMATION

Funding was provided by the Great Lakes Fishery Commission (via a BOTE Grant to SJC) and by the GenFish program of Genome Canada (via Grant to SJC).
